# Diversity of glutamate receptors in glial cells of the mouse optic nerve

**DOI:** 10.3389/fncel.2026.1911068

**Published:** 2026-09-08

**Authors:** Nine F. Kompier, Marcus Semtner, Sophie Walter, Christian Steinhäuser, Christiane Nolte, Helmut Kettenmann

**Affiliations:** 1 Max-Delbrück-Center for Molecular Medicine in the Helmholtz, Cellular Neurosciences Group, Berlin, Germany; 2 Social Neuroscience Lab, Max-Planck-Gesellschaft, Berlin, Germany; 3 Experimental Ophthalmology, Charité Universitätsmedizin, Campus Virchow, Berlin, Germany; 4 Institute for Biology, Free University Berlin, Berlin, Germany; 5 Medical Faculty, Institute of Cellular Neurosciences I, University of Bonn, Bonn, Germany; 6 Bio-X International Institute, Shenzhen University of Advanced Technology, Shenzhen, China

**Keywords:** NG2 glia, astrocytes, glutamate receptors, optic nerve, patch-clamp

## Abstract

**Introduction:**

Glial cells in the optic nerve express ionotropic glutamate receptors that may contribute to axon–glia communication, yet their functional expression in defined glial populations remains incompletely characterized. We investigated glutamate receptor-mediated responses in identified NG2 glia and astrocytes of the mouse optic nerve.

**Methods:**

We used a longitudinal optic nerve slice preparation combined with whole-cell patch-clamp recordings. NG2 glia and astrocytes were identified and electrophysiologically characterized using the transgenic reporter mouse lines hGFAP-eGFP and Cx43-ki-eCFP, respectively. Functional responses to kainic acid (KA), activating AMPA/kainate receptors, and NMDA were assessed at the single-cell level.

**Results:**

KA evoked membrane current responses in the majority of NG2 glia, whereas NMDA responses were restricted to a smaller subset and were associated with the absence of A-type K+ currents. In passive astrocytes, approximately half of the cells responded to KA and NMDA. KA responses showed heterogeneous current patterns, including increased cationic conductance and, in a subset of cells, reduced outward K+ currents, whereas NMDA responses were comparatively homogeneous.

**Discussion:**

These findings demonstrate substantial functional heterogeneity of ionotropic glutamate receptor responses within established optic nerve glial populations. Glutamate responsiveness does not define distinct glial cell types but rather represents an additional functional feature within NG2 glia and astrocytes. This diversity may enable glial cells to adapt to local neuronal activity and contribute to axon–glia communication and white matter homeostasis.

## 1 Introduction

Macroglial cells in the central nervous system (CNS)—astrocytes, oligodendrocytes, and NG2-glia—play key roles in maintaining homeostasis and modulating neuronal activity under both physiological and pathological conditions (Allen and Lyons, 2018; Kettenmann et al., 2025; McNeill et al., 2021). Among these, NG2 cells represent a specialized glial population originally considered as oligodendrocyte precursor cells. While many NG2 cells retain oligodendrocyte precursor cell (OPC) properties, they also persist as a separate cell population throughout life, suggesting additional roles beyond oligodendrogenesis (Dimou et al., 2008; Kang et al., 2010; Trotter et al., 2010). Indeed, they can receive synaptic input from neurons (Bergles et al., 2010). These cells are characterized by the expression of the chondroitin sulfate proteoglycan NG2 (Butt et al., 2005; Butt et al., 2002; Nishiyama et al., 2005).

NG2 cells and astrocytes can be distinguished by their electrophysiological properties. Astrocytes typically display linear membrane currents lacking voltage-dependent kinetics, resting membrane potentials close to the K^+^ equilibrium potential and low input resistance (Bedner et al., 2020; Matyash and Kettenmann, 2010; Steinhauser et al., 1992). In contrast, NG2 cells express a range of voltage-gated channels, including prominent A-type K^+^ currents, resulting in more complex membrane current profiles (Bedner et al., 2020). These distinctions were recently confirmed in the rodent optic nerve (Kompier et al., 2024), a widely used white matter tract model to study axon–glia interactions and CNS energy metabolism (Brown et al., 2019; Butt et al., 2004). Using transgenic reporter mice and a novel acute nerve preparation allowing patch-clamp recordings from identified cells, we showed that optic nerve astrocytes (identified in Cx43-ki-eCFP mice) are predominantly passive, although a small electrophysiologically distinct subset, called type 2 cells, exhibit additional leak-like and minor voltage-dependent conductance (Kompier et al., 2024). In contrast, NG2 cells—identified in NG2-ki-eYFP and hGFAP-eGFP mice—display complex membrane properties, including A-type K^+^ currents in the majority of cells. Interestingly, although hGFAP-eGFP mice are commonly used to label astrocytes (Nolte et al., 2001), in the optic nerve, eGFP expression is predominantly found in NG2-positive/GFAP-negative cells with complex electrophysiological profiles. These cells, initially termed ON type 1 cells based on their electrophysiological properties, resembled NG2 cells identified in NG2-ki-eYFP mice (Kompier et al., 2024). They were consistently NG2+, and thereby considered NG2-glia. A small subset of hGFAP-eGFP+ cells displayed more passive/leak-like or weakly voltage-dependent currents resembling the so-called type 2 cells identified in Cx43-ki-eCFP mice. As these cells were consistently NG2-negative, they were considered to be likely astrocytes. Together, these findings indicate that the optic nerve contains electrophysiologically complex NG2 cells and probably at least two astrocyte subtypes with distinct membrane properties.

Both NG2 cells and astrocytes express functional neurotransmitter receptors, including ionotropic glutamate receptors, enabling them to sense local neuronal activity. Electrophysiological studies have demonstrated the presence of AMPA/kainate and NMDA receptors on macroglial cells across multiple CNS regions (Ceprián Costoso and Fulton, 2019; Hogan-Cann and Anderson, 2016), suggesting a role in mediating glial responses to synaptic and axonal signaling. Importantly, receptor expression patterns vary between cell types and depend on regional context, particularly between gray and white matter (Köhler et al., 2021; Steinhäuser and Dietrich, 2015). However, data on neurotransmitter receptor function in optic nerve glia remain limited. Earlier studies primarily relied on Ca^2+^ imaging in whole-nerve explants (Butt et al., 2004), identifying glial cells based on morphology rather than molecular markers. Only recently, the development of an optic nerve longitudinal slice preparation enabled the application of patch-clamp recordings in this preparation and enabled us to characterize ionotropic glutamate receptor-mediated membrane currents from glial cells in this tissue (Kompier et al., 2024). Transgenic reporter mice enabled us to identify cells as astrocytes (Cx43-ki-eCFP) or NG2-glia (hGFAP-eGFP). In the latter, we specifically analyzed cells with complex membrane properties (type 1), previously shown to correspond to NG2-glia based on immunohistochemistry. Using this approach, we investigated glutamate receptor-mediated responses to KA and NMDA at the single-cell level. We used KA at a concentration of 0.1 mM; at this concentration it activates both kainate- and AMPA-type glutamate receptors (Jane, 2007; Lee et al., 2004; Seifert and Steinhäuser, 1995; Tomita et al., 2007). The aim of the present study was to distinguish between functional responses evoked by NMDA and the other ionotropic glutamate receptor subtypes, namely kainate and AMPA. Our results reveal pronounced heterogeneity in both the prevalence and kinetics of glutamatergic responses in NG2 cells and astrocytes, highlighting the functional complexity of macroglial populations in ON.

## 2 Methods

### 2.1 Animals and handling

Mice were housed in the local animal facility of the Max Delbrück Center, Berlin under a 12 h/12 h light–dark cycle with food and water provided ad libitum. For experiments on NG2-glia, we used hGFAP-eGFP mice (Hirrlinger et al., 2005; Matthias et al., 2003; Nolte et al., 2001), in which we previously identified two populations (Kompier et al., 2024): type 1 cells, which show complex electrophysiological characteristics resembling NG2-glia. Based on immunohistochemistry, these cells were confirmed to correspond to NG2-glia. A second population, type 2 cells, showed more passive, leak-dominated conductance, and were consistently NG2-negative. These cells were considered astrocyte-like (Kompier et al., 2024). For experiments in NG2-glia, therefore, only type 1 cells displaying membrane properties characteristic of NG2 cells were included in the analysis. Type 2 cells were analysed as a separate class of likely astrocytes. For astrocyte experiments, we used Cx43-ki-eCFP transgenic mice, in which one allele of the gap junction protein Cx43 (Gja1) is replaced by eCFP (Degen et al., 2012), reliably labeling astrocytes in the optic nerve (Kompier et al., 2024). Two different electrophysiological populations can be identified among the Cx43-ki-eCFP: cells with completely passive membrane currents, typical for astrocytes, and a population previously identified in hGFAP-eGFP mice with only weakly voltage-dependent, leak dominated currents. Both mouse strains were on a C57BL/6 background, of both sexes, and aged 4–8 weeks.

### 2.2 Immunostaining and microscopy

To visualize glial cell populations in the optic nerve, immunohistochemistry was performed on acute slices. Tissue was fixed in 4% paraformaldehyde in 0.1 M phosphate-buffered saline (PBS, pH 7.4) overnight at 4 °C. After washing, samples were incubated in blocking solution containing 2% Triton X-100, 2% bovine serum albumin (BSA), and 8% normal donkey serum (NDS) in Tris-buffered saline (TBS) for 2–3 h at room temperature. Primary antibodies were diluted in TBS containing 1% Triton X-100, 2% BSA, and 8% NDS and applied for 48 h at 4 °C. The following primary antibodies were used: guinea pig anti-GFAP (1:500, Synaptic Systems), and chicken anti-GFP (1:400, Abcam) to enhance transgenic fluorescence signals for eGFP (hGFAP-eGFP) and eCFP (Cx43-ki-eCFP). After washing in TBS-T (TBS containing 0.1% BSA and 0.5% Tween), samples were incubated with appropriate secondary antibodies (Alexa Fluor-conjugated donkey antibodies, 1:200; Jackson ImmunoResearch) for 2 h at room temperature. Nuclei were counterstained with DAPI (50 ng/mL). Samples were mounted using Aqua Poly/Mount (Polysciences). Control experiments omitting primary antibodies showed no unspecific staining.

Fluorescent images were acquired using a Zeiss LSM 700 confocal microscope controlled by ZEN software. Z-stacks were obtained with 1 μm step size using a 40x oil immersion (NA 1.3) objective, with the pinhole set to 1 Airy unit. Images were processed using Fiji (ImageJ) for visualization and maximum intensity projections.

### 2.3 Preparation of acute optic nerve slices

For preparation of acute optic nerve slices, we used a longitudinal optic nerve slice preparation first described in Kompier et al. (2024). Briefly, 4–8 week-old transgenic mice were sacrificed by cervical dislocation and their optic nerves were removed by gently removing the eye from the orbit using forceps. Nerves were separated from the eyes at the base under a dissection microscope (Olympus SZ40s, Hamburg, Germany), and placed at the bottom of a petri dish filled with 5% low-melting agarose (Bio&SELL, Feucht, Germany, BS20.47.025) in Hank’s balanced salt solution (HBSS; Gibco, Thermo Fisher, Inc. Waltham, MA, USA, 14025092) supplemented with Ca^2+^ and Mg^2+^. Temperature at the time of embedding was 25 °C. After gelling of the agarose, a square holding the optic nerve was cut, and mounted in a chamber with ice-cold bicarbonate-buffered slicing solution composed of 230 mM sucrose, 26 mM NaHCO_3_, 2.5 mM KCl, 1.25 mM NaH_2_PO_4_, 10 mM MgSO_4_, 0.5 mM CaCl_2_, and 10 mM glucose; pH 7.4. The solution was continuously gassed with carbogen (95% O_2_, 5% CO_2_). On a vibratome (HM 650 V, Microm International GmbH, Walldorf, Germany), longitudinal optic nerve slices of 150 μm were prepared at 4 °C. Slices were stored at room temperature (21 °C) for up to 5 h in artificial cerebrospinal fluid (ACSF) composed of 134 mM NaCl, 2.5 mM KCl, 1.3 mM MgCl_2_, 2 mM CaCl_2_, 1.26 mM K_2_HPO_4_, 26 mM NaHCO_3_ and 10 mM glucose; pH 7.4. ACSF was continuously gassed with carbogen (95% O_2_, 5% CO2).

### 2.4 Patch-clamp recordings of basic membrane properties

Basic membrane currents were recorded to confirm cellular identity and assess recording quality. Experiments were performed as previously described (Kompier et al., 2024; Richter et al., 2014). Slices were transferred to a recording chamber mounted on an upright microscope (Slicescope II, Scientifica Ltd., Uckfield, UK) equipped with a 60 × water immersion objective (Olympus, Hamburg, Germany) and secured using a U-shaped, in-house-built platinum grid. The chamber was continuously perfused with ACSF using a peristaltic pump (Minipuls 3, Gilson, Middleton, USA). EGFP fluorescence in hGFAP-eGFP mice was detected using excitation/emission wavelengths of 488/510 ± 10 nm, and eCFP fluorescence in Cx43-ki-eCFP mice at 433/475 ± 10 nm. Patch pipettes (4–8 MΩ) were pulled from borosilicate glass (1.5 mm outer diameter, 0.315 mm wall thickness) and filled with intracellular solution (ICS) containing 100 mM K-gluconate, 30 mM KCl, 10 mM glucose, 1 mM MgCl₂, 0.5 mM CaCl₂, 10 mM HEPES, 5 mM EGTA, 0.5% biocytin, and 3 mM Na₂ATP (pH 7.3). Sulforhodamine B (10 μg/mL; Sigma-Aldrich) was added to monitor intracellular solution exchange and confirm successful whole-cell access. Fluorescence illumination (586 ± 10 nm) was used during recordings to assess dye diffusion and after transmitter application to verify that the recorded cell remained filled.

Membrane currents were evoked by voltage steps from a holding potential of −70 mV ranging from −160 to +50 mV (100 ms duration; 10 mV increments), using an EPC 10 patch-clamp amplifier and TIDA 5.25 software (HEKA Elektronik). Pipette capacitance (C_fast_) was compensated online, whereas membrane capacitance and series resistance (C_slow_) were not compensated. Reversal potentials were corrected for the liquid junction potential (−8.9 mV), calculated using Patcher’s Power Tools and IGOR Pro 7. A-type potassium currents, often present in NG2 cells, but not in astrocytes, were assessed using a protocol adapted from Sontheimer et al. (1989). Currents were evoked by depolarizing steps from −60 to +20 mV (50 ms; 10 mV increments) from holding potentials of −70 mV and −110 mV. Subtraction of the resulting currents at corresponding voltages revealed fast inactivating A-type K^+^ currents.

### 2.5 Neurotransmitter application

After recording basic membrane currents and A-type K^+^ currents, KA and NMDA were applied to characterize functional ionotropic glutamate receptor expression in hGFAP-eGFP and Cx43-ki-eCFP mice. Cells were clamped at a holding potential of −70 mV in voltage clamp mode, and a series of de- and hyperpolarizing voltage steps ranging from −100 to 60 mV for 50 ms each was applied with 20 mV increments to determine membrane conductance. This protocol was repeated every 5 s to continuously monitor membrane currents upon substance application. After perfusing the recording chamber (4–6 mL/min) with standard recording ACSF for 2 min, bath perfusion of normal ACSF was supplemented with 100 μM NMDA or 100 μM KA for 30 s. Order of neurotransmitter application was randomized and separated by a 3-min wash-out period with normal ACSF. In neurons, NMDA receptors are double-gated through depolarization-dependent removal of a Mg^2+^-block upon sufficient depolarization. In astrocytes, responses to NMDA are insensitive or only weakly sensitive to Mg^2+^ (Káradóttir et al., 2005; Lalo et al., 2006; Palygin et al., 2011; Richter et al., 2014). To test sensitivity to Mg^2+^ in NG2-glia, responses to NMDA were tested by supplementing Mg^2+^-free ACSF for at least 3 min and then adding 100 μM NMDA for 30 s.

### 2.6 Data analysis

All single-cell electrophysiological recordings were processed using an in-house written software in IGOR Pro 6.37 (WaveMetrics; RRID:SCR_000325) (Wendt et al., 2017). IV curves were calculated and the following properties of each cell at start and end of the dialysis were determined: series resistance, membrane resistance, membrane capacitance and reversal potential. To that end, membrane currents were averaged for quantification between 50 and 95 ms after clamping the membrane to a given value from the resting potential. Membrane capacitance was calculated based on an exponential fit of the current decay in response to a 10 ms test pulse. The same pulse was used to quantify the series resistance from the peak amplitude of the membrane capacitance currents (Wendt et al., 2017). Basic membrane properties were used to determine whether non-responding and responding cells, or cells with different response dynamics, showed different membrane properties. To determine the conductance of the neurotransmitter response, IV curves were calculated right before (baseline) and after neurotransmitter application as previously described (Wendt et al., 2017).

### 2.7 Experimental design and statistical analysis

Igor Pro 6.37 (WaveMetrics; RRID:SCR_000325) and GraphPad Prism 7 (RRID:SCR_002798) were used for statistical analysis. Results in the text and graphs are reported as median (25/75 percentile), unless otherwise stated; *N* = number of mice; *n* = number of cells/brain slices. Statistical tests are described in the figure legends, and were applied to compare basic membrane properties and/or presence of A-type K^+^ currents between responding and non-responding cells. Normal distribution for all data was tested using the D’Agostino-Pearson test. Data comparing two independent groups were analyzed using two-sided Mann–Whitney U tests due to non-parametric distributions. Statistical significance was defined as *p* < 0.05 (*)*, p <* 0.01(**), *p <* 0.001(***), or *p* < 0.0001 (****). Where multiple variables were tested, multiplicity-adjusted *p*-values (Bonferroni-type correction) are reported in the figure legends to account for multiple comparisons. Categorical data were analyzed using chi-square (*χ*^2^) tests of independence to assess associations between group classifications (e.g., responding vs. non-responding) and categorical outcomes (e.g., presence or absence of ion channel currents). Where appropriate, Fisher’s exact test was used instead for small sample sizes.

## 3 Results

We have previously shown that GFAP-eGFP+ cells in the optic nerve (ON) of hGFAP-eGFP transgenic mice do not represent astrocytes as in other brain regions, but mainly label a glial population with NG2-like properties (Kompier et al., 2024). Patch-clamp recordings revealed two electrophysiologically defined populations: a major group of cells (ON type 1) exhibiting complex time- and voltage-dependent membrane currents, including prominent outward currents and with a subset displaying A-type potassium currents. A smaller group (ON type 2) is characterized by predominantly leak-like membrane currents with only weak voltage-dependent components and could be clearly distinguished electrophysiologically from ON type 1 cells. Previously, we have shown that electrophysiological ON type 1 cells are consistently NG2+, and are thereby considered NG2-glia. The electrophysiological subtype ON type 2 were consistently NG2-, however, their cellular identity could not be established (Kompier et al., 2024).

**Figure 1:**
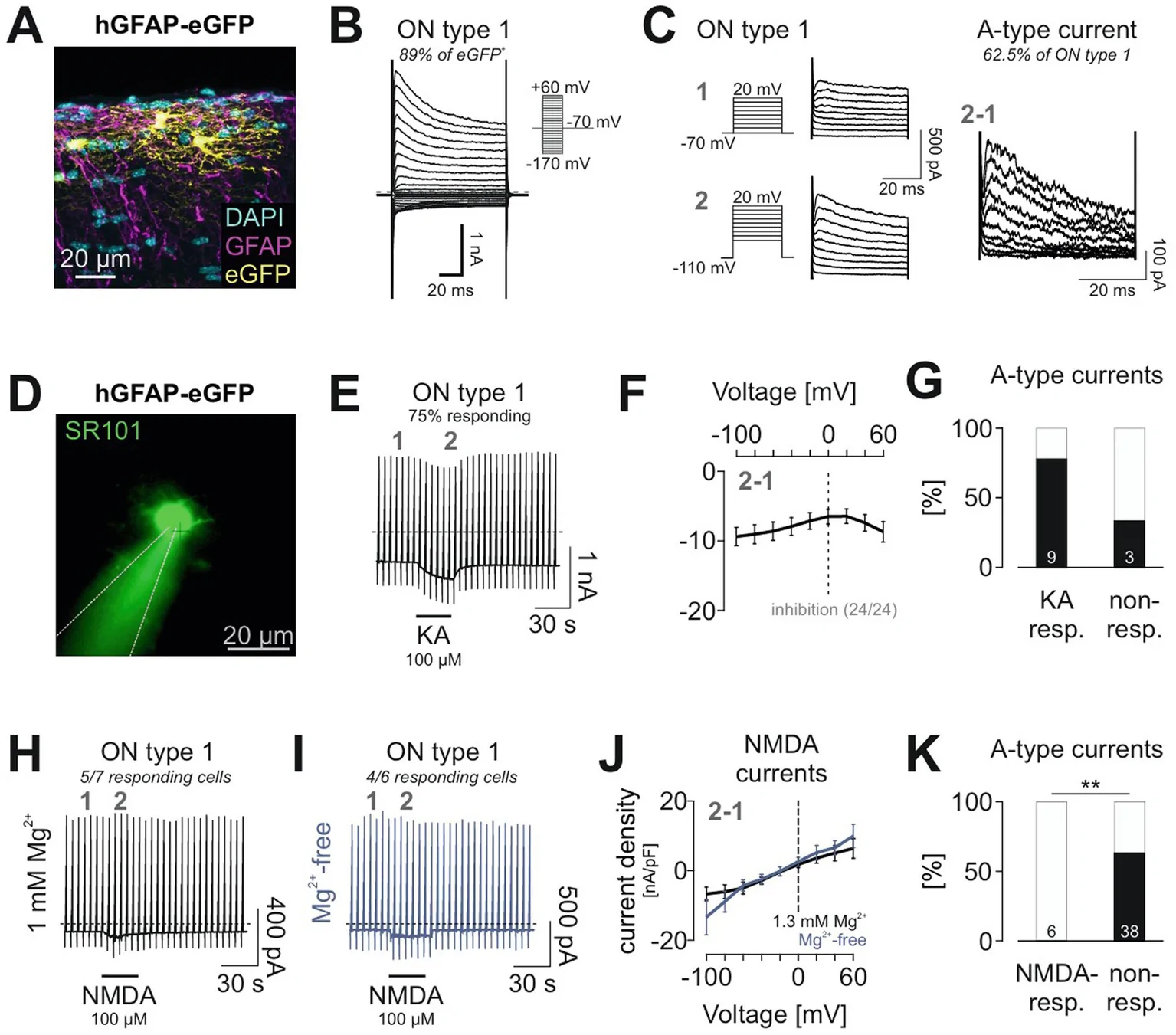
Electrophysiological identification of ON type 1 cells and their responses to KA and NMDA. **(A)** Confocal image of the optic nerve (ON) from a hGFAP-eGFP mouse showing eGFP-expressing cells (yellow), GFAP immunostaining (magenta), and DAPI-labeled nuclei (blue). Scale bar: 20 μm. **(B)** Representative membrane currents of ON type 1 cells in response to voltage steps (−170 mV to +60 mV), illustrating their characteristic time- and voltage-dependent current profile. ON type 1 cells represent the majority (~89%) of eGFP+ cells. **(C)** Detection of A-type potassium currents using a standard voltage protocol (holding potentials −70 mV and −110 mV). Subtraction (2–1) reveals fast inactivating A-type K^+^ currents, present in a subset of ON type 1 cells (62.5%). **(D)** Representative image of a patched eGFP+ cell filled with SR101. The dotted outline indicates the patch-pipette. Scale bar: 20 μm. **(E)** Representative current trace showing the response of an ON type 1 cell to KA (100 μM, 30 s). Each vertical line represents a series of voltage steps (10 mV increment) from a holding potential of −70 mV. The response is characterized by a simultaneous decrease in outward current and increase in inward current. **(F)** Corresponding current–voltage (IV) relationship derived from recording shown in **(E)**. The current values are for baseline (1) and peak response (2), illustrating the absence of a clear reversal potential. **(G)** Quantification of the presence of A-type K^+^ current in KA-responding and non-responding cells (as percentage, black: Presence of A-type K^+^ current, white: No A-type K^+^ current). Numbers in the bars represent the absolute number of cells displaying A-type K^+^ currents. **(H,I)** Representative current traces showing NMDA-evoked responses (100 μM, 30 s) in ON type 1 cells under control (1.3 mM Mg^2+^; H) and Mg^2+^-free **(I)** conditions. **(J)** Current density-voltage relationships of NMDA-induced currents under control (1.3 mM Mg^2+^, black) and Mg^2+^-free (blue) conditions, showing near-linear current–voltage relationship and a reversal potential close to 0 mV. **(K)** Distribution of A-type K^+^ currents in NMDA-responding and non-responding cells, demonstrating that NMDA responses are restricted to cells lacking A-type K^+^ currents. The proportion of A-type vs. no-A-type differed significantly between NMDA responding and non-responding cells (Fisher’s exact test, *n* = 44). Note that experiments under control (1.3 mM Mg^2+^) and Mg^2+^-free conditions were pooled in this panel.

We confirmed our previous findings by patch clamp experiments and found that the majority of recorded cells (89% of *n* = 47 cells) displayed complex membrane current profiles characterized by time- and voltage-dependent conductance (Figures 1A,B), high input resistance (118.6 (89.4/192.2) MΩ, *n* = 42 cells from *N* = 25 mice) and less negative membrane potentials (−64.7 (−74/−54.7) mV), consistent with ON type 1 cells as previously described. In addition, A-type potassium currents were detected in a substantial fraction of these cells (10 out of 16 cells, 62.5%) (Figure 1C). Importantly, the presence or absence of an A-type potassium current was assessed within the ON type 1 population and does not define the distinction between ON type 1 and ON type 2 cells. Cells lacking detectable A-type currents retained the characteristic complex membrane current profile of ON type 1 cells. A smaller population of GFAP-eGFP+ cells exhibited predominantly leak-like currents with weak voltage dependence, consistent with astrocyte-like properties as described previously (Supplementary Figures S1A–C). This population was distinct from the more passive electrophysiological profile observed in Cx43-ki-eCFP-positive astrocytes. Thus, in accordance with our previous findings, GFAP-eGFP+ cells in the optic nerve comprise two electrophysiologically distinct populations, with the majority corresponding to NG2-glia-like (ON type 1, Supplementary Figure S1D) and a minority representing ON type 2 cells, which, based on previous immunohistochemical results, are considered NG2-. Because the cellular identity of type 2 cells could not be established, these cells were not included in the NG2-glia analysis and were considered separately (Supplementary Figures S1A–C).

**Figure 2:**
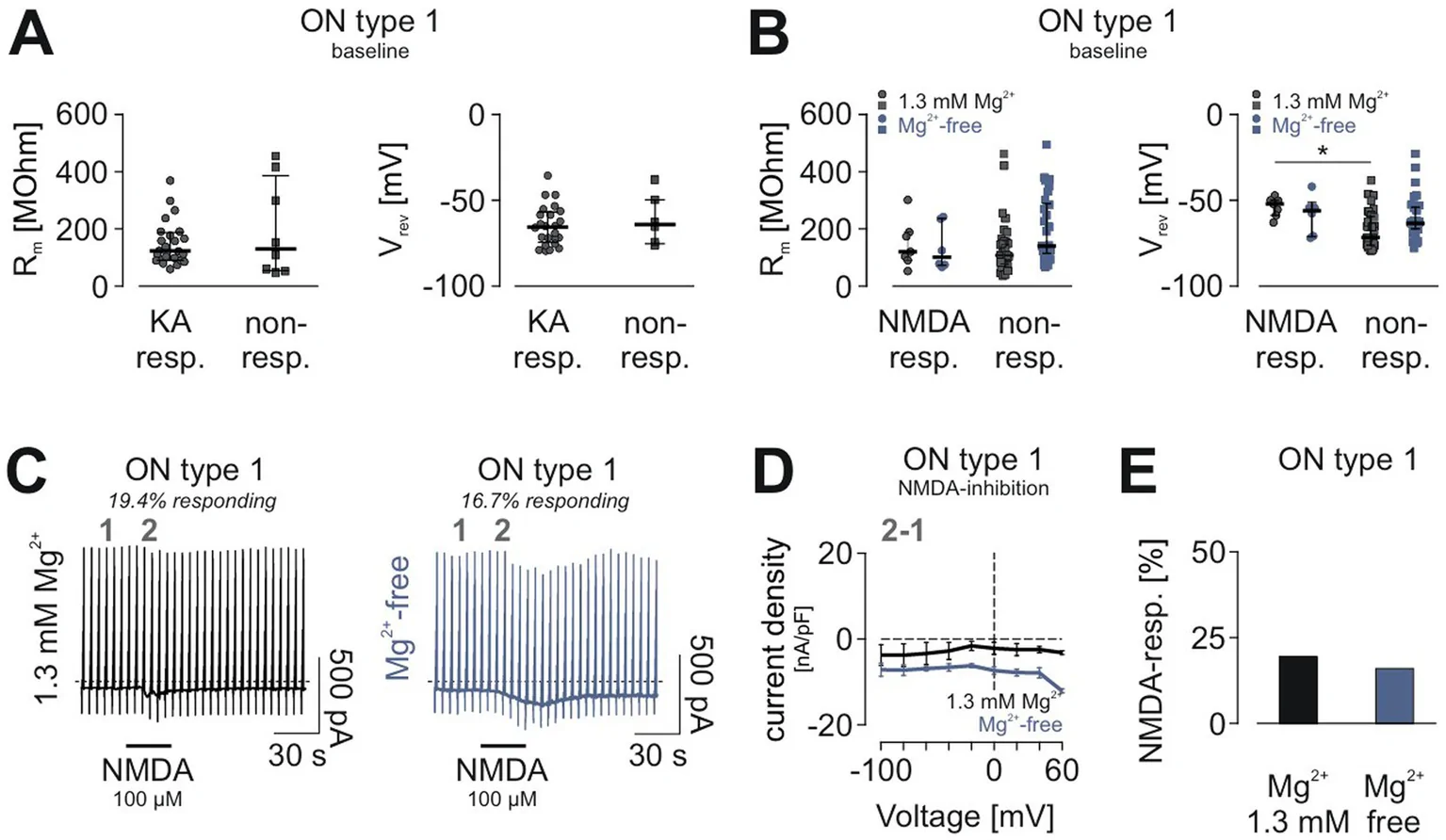
Glutamate responses in ON type 1 glia. **(A)** Quantification of membrane resistance (left) and reversal potential (right) showing no differences in KA-responding and non-responding ON type 1 cells. **(B)** Quantification of membrane resistance (left) and reversal potential (right) in NMDA-responding and non-responding ON type 1 cells under Mg^2+^-containing (1.3 mM) and Mg^2+^-free conditions. **(C)** Representative current traces showing responses of an ON type 1 cell to NMDA (100 μM, 30 s) under Mg^2+^-containing (1.3 mM; left) and Mg^2+^-free conditions (right). The response is characterized by a simultaneous decrease in outward current and increase in inward current. **(D)** Current density-voltage relationships of NMDA-induced currents under control (1.3 mM Mg^2+^, black) and Mg^2+^-free (blue) conditions, illustrating the absence of a clear reversal potential. **(E)** Fraction of NMDA-responsive ON type 1 cells in standard and Mg^2+^-free conditions. Both types of responses (increasing and decreasing outward currents) were taken into account.

As glutamate release from axons has been suggested to provide a mechanism for axon–glia signaling, we tested whether ON type 1 cells exhibit KA-mediated receptor responses. Application of KA (100 μM; Figures 1D–F) for 30 s induced membrane conductance changes in the majority of the investigated cells. Specifically, 75% of ON type 1 cells (24 out of 32 cells from *N* = 21 mice) responded to KA. In all responding cells, the current response was characterized by a simultaneous decrease in outward current and an increase in inward current, developing over a similar time course during agonist application (Figure 1E). This response pattern was consistently observed across cells and resembled previously described KA-induced currents in optic nerve or hippocampal NG2 cells *in situ* or *in vitro* (Borges et al., 1995; Schröder et al., 2002). Due to the dual effect on inward and outward conductance, the resulting IV relationships lacked a clear reversal potential (Figure 1F). KA sensitivity did not correlate with any baseline electrophysiological properties, i.e., there was no difference between KA responding and non-responding ON type 1 cells with regard to their baseline membrane resistances (*p* = 0.9490) or membrane potentials (*p* = 0.6462; *n* = 32 cells, Figure 2A). There was no correlation between the population of cells expressing A-type K^+^ currents (A-type K^+^ currents in 7 out of 9 tested cells) and the population responding to KA (Figure 1G).

We next asked whether ON type 1 cells exhibit NMDA receptor responses and whether these responses are uniformly distributed across this glial population. In contrast to KA, NMDA application (100 μM) for 30 s elicited membrane current responses in only a minority of ON type 1 cells, i.e., 19.4% (7 out of 36 cells from *N* = 23 mice; Figure 1H). In the majority of responding cells (5 out of 7), NMDA induced a linear increase in both inward and outward conductance, resulting in near-linear IV relationships with a reversal potential of −12.2 mV (Figure 1J), consistent with activation of a non-selective cation current. In a smaller subset of responding cells (2 out of 7), NMDA-evoked currents resembled those observed following KA application, characterized by a simultaneous reduction of outward current and an increase in inward current (Figures 2C,D). Thus, while most NMDA responses were consistent with NMDA receptor activation, a minority displayed atypical response profiles. NMDA-responding cells did not significantly differ from non-responding cells in terms of membrane resistance (*p* = 0.5629), but had a significantly less negative membrane potential (−51.36 (−58.04/−49.2) mV vs. -70.97 (−75.53/−58.67) mV, *p* = 0.005) (Figure 2B). As NMDA receptor function is typically voltage- and Mg^2+^-dependent, we tested whether NMDA responses in NG2 cells are modulated by extracellular Mg^2+^. Removal of Mg^2+^ from the extracellular solution did not alter the baseline electrophysiological properties of ON type 1 cells. Membrane resistance (143.9 (106.4/270.2) MΩ, *p* = 0.072) and reversal potential (−65.9 (−69.7/−56.9) mV, *p* = 0.9464) remained unchanged compared to standard conditions (Supplementary Figures S2A,B). In addition, the proportion of cells expressing A-type K^+^ currents (16 out of 28 cells, 57%) was unaffected by Mg^2+^ removal (Supplementary Figure S2C), suggesting that NMDA receptors contribute little, if at all, to ON type 1 membrane properties at baseline. Accordingly, NMDA responsiveness was not enhanced under Mg^2+^-free conditions. The fraction of responding cells remained low (16.7%, 6 out of 36 cells from *N* = 18 mice), comparable to that observed in standard ACSF (Figures 1H–J, 2E). In responding cells, NMDA-induced currents again showed a linear IV relationship with a reversal potential of −20.2 mV (4 out of 6 cells), while a minority of cells (2 out of 6) displayed atypical responses characterized by a reduction in outward current and an increase in inward current (Figures 2C,D). As removal of Mg^2+^ did not affect baseline membrane properties or NMDA responsiveness, data from Mg^2+^-containing and Mg^2+^-free conditions were pooled. This revealed a clear association with electrophysiological subtype: NMDA responses were exclusively observed in cells lacking A-type K^+^ currents (6/6 responding cells), whereas the majority of non-responding cells expressed A-type K^+^ currents (24/38 cells, 63%). This distribution differed significantly between groups (Fisher’s exact test, *p* = 0.005; *n* = 44; Figure 1K). Together, these results indicate that NMDA receptor function in NG2 cells is independent of extracellular Mg^2+^ but is restricted to a specific electrophysiological subclass defined by the absence of A-type potassium currents.

**Figure 3:**
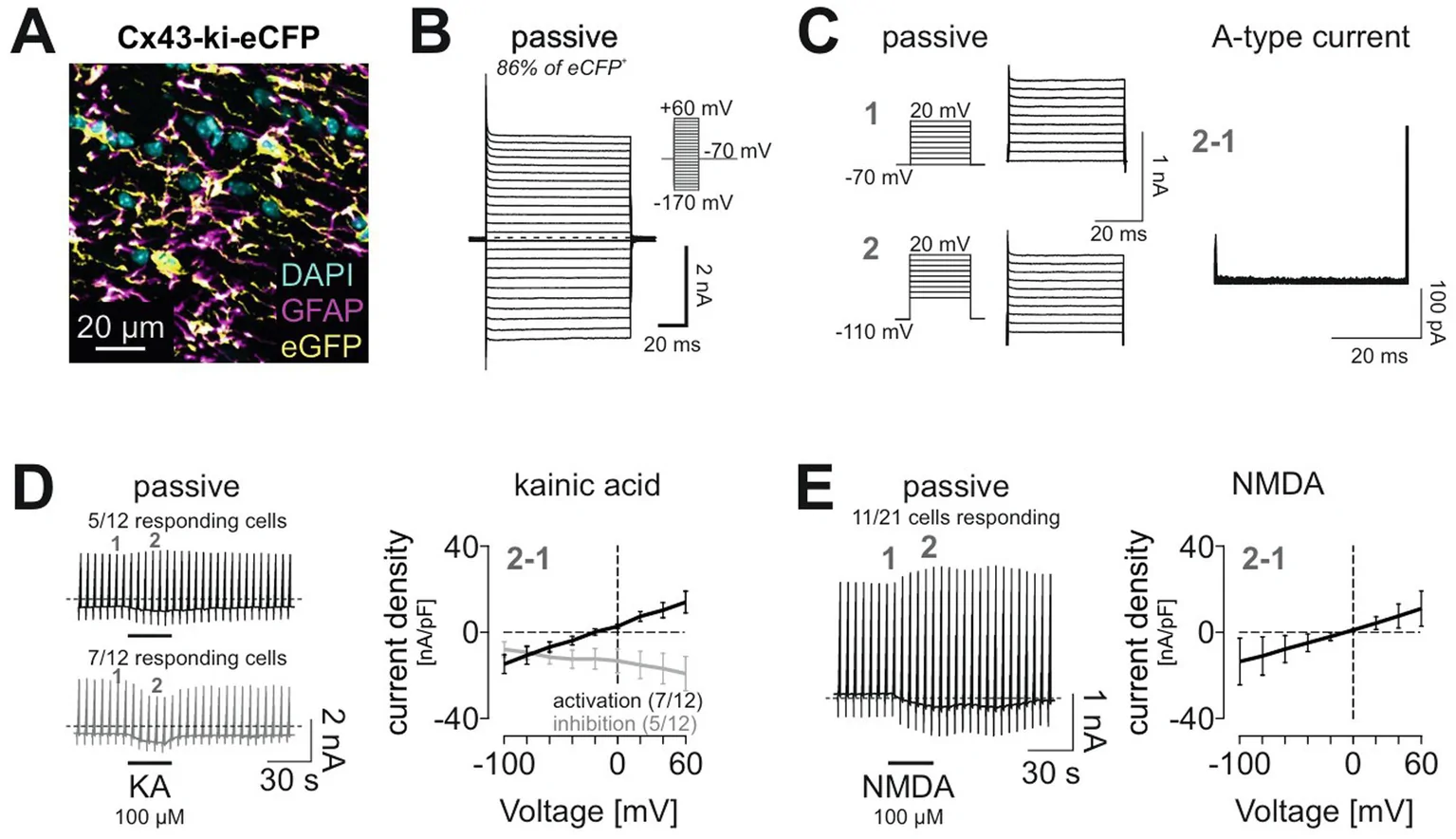
Astrocyte subtype-specific responses to KA and NMDA. **(A)** Confocal image of the optic nerve from a Cx43-ki-eCFP mouse showing astrocytes (eCFP, yellow), GFAP (magenta), and nuclei (DAPI, blue). Scale bar: 20 μm. **(B)** Representative membrane currents of passive astrocytes, displaying linear current–voltage relationships and lacking voltage-dependent components. Passive cells constitute the majority (~86%) of eCFP+ cells. **(C)** Representative voltage protocol demonstrating the absence of A-type potassium currents in ON passive cells after applying the protocol described in the legend to Figure 1C. **(D)** Representative current traces as described in the legend of Figure 1E (*left*) and corresponding current density-voltage relationships (*right*) showing responses of passive astrocytes to KA (100 μM, 30 s). Two response types were observed: increased cationic conductance (7/12 cells) and reduction of outward current (5/12 cells). Overall, 52.2% of ON passive cells responded. **(E)** Representative current traces and IV relationships showing NMDA-induced responses (100 μM, 30 s) in ON passive cells. NMDA responses were homogeneous and characterized by increased inward and outward conductance with a reversal potential close to 0 mV (52.4% responding).

We next examined functional glutamate receptor responses in astrocytes and asked whether these responses also differ between electrophysiologically defined subtypes. Astrocytes were identified using the Cx43-ki-eCFP mouse line (Degen et al., 2012) and classified based on their membrane current profiles into ON passive cells (Figures 3A,B). Most eCFP-positive cells displayed a largely passive membrane current profile characteristic of astrocytes in gray matter brain structures like cortex or hippocampus (Kompier et al., 2024). In addition, and consistent with our previous report (Kompier et al., 2024) a smaller population of eCFP-positive cells exhibited leak-dominated currents with weak voltage-dependent components resembling the ON type 2 phenotype identified in hGFAP-eGFP mice (Supplementary Figure S3A). Because of the limited number of these cells in both mouse lines, we did not further characterize this population.

As shown in Figure 3C, Cx43^+^ passive cells did not express A-type K^+^ currents. KA application (100 μM, 30 s) elicited membrane current responses in 52% of passive astrocytes (12 out of 23 cells from *N* = 15 mice, Figures 3D,E). In the majority of responding cells, KA induced a linear increase in membrane conductance with a reversal potential of −11.5 mV, consistent with activation of a cationic current (Figure 3D). In a subset of cells (5 out of 12 responders), KA responses were instead characterized by a reduction of outward current (Figure 3D), resembling the response pattern observed in NG2-like, ON type 1 glia. However, neither membrane resistance (*p* = 0.2805) nor reversal potential (*p* = 0.5765) predicted KA responsiveness among passive astrocytes (Supplementary Figure S3B).

NMDA application (100 μM, 30 s) evoked membrane current responses in 52% of passive astrocytes (11 out of 21 cells from *N* = 15 mice), a markedly higher proportion than observed in NG2-glia. In all responding cells, NMDA induced a uniform increase in inward and outward conductance, resulting in near-linear IV relationships with a reversal potential close to zero (−8.8 mV), consistent with activation of NMDA receptor-mediated currents (Figure 3E). In contrast to ON type 1, NMDA responses in passive astrocytes were homogeneous in their kinetics and were not associated with baseline membrane properties, including membrane resistance (*p* = 0.4700) and reversal potential (*p* = 0.46, Supplementary Figure S3C). Together, these results show that ON passive cell responses to glutamate receptor activation are more uniform than in NG2-glia, with robust and consistent KA and NMDA responses.

## 4 Discussion

In the present study, we used a recently developed longitudinal optic nerve slice preparation in combination with transgenic reporter mice to assess responses to NMDA and KA in identified astrocytes and NG2-glia. We should emphasize that KA at the concentration used in our study activates both kainate- and AMPA-subtypes of glutamate receptors as mentioned in the introduction. Thus, our KA activated responses can be attributed to both, AMPA and kainate receptors. Our data reveal a substantial degree of functional heterogeneity in both cell types, reflected by variable responsiveness to KA and NMDA. Importantly, glutamate receptor responses did not define distinct glial cell classes, but rather occurred in a subset of cells within the previously established major populations. Thus, receptor responsiveness appears to represent an additional functional layer within defined glial subtypes rather than a primary classification criterion. These findings extend our previous electrophysiological characterization of macroglial populations in the optic nerve (Kompier et al., 2024) and demonstrate that subsets of both NG2 cells and astrocytes express functional ionotropic glutamate receptors. The variability in response prevalence and kinetics suggests that glutamatergic signaling in white matter glia is more heterogeneous and dynamic than previously appreciated, potentially supporting diverse roles in axon–glia communication and metabolic coupling.

KA induced membrane currents in the majority of NG2 cells (ON type 1) in the optic nerve, indicating widespread functional expression of KA-sensitive receptors. Similar response rates have been reported in NG2 cells from other CNS regions, including hippocampus (Jabs et al., 1994; Steinhauser et al., 1994). The characteristic response, an increase in inward conductance accompanied by a reduction of outward K^+^ conductance, closely resembles previously described KA-mediated effects in NG2 cells both *in situ* and *in vitro* (Borges et al., 1995; Schröder et al., 2002). This reduction in outward K^+^ conductance is likely mediated by Na^+^ influx through the receptor channel (Borges and Kettenmann, 1995) and has been proposed to contribute to limiting extracellular K^+^ accumulation during neuronal activity. Although the functional role of KA-activated receptors on NG2 cells in the optic nerve remains incompletely understood, glutamate release from axons, particularly at nodes of Ranvier, has been shown to trigger Ca^2+^ signaling in NG2 cells (Ceprián Costoso and Fulton, 2019). This pathway may contribute to activity-dependent regulation of proliferation, differentiation, and myelination (Cherchi et al., 2021; Gallo et al., 2008; Gautier et al., 2015; Larson et al., 2016). Notably, KA responses were observed in the majority of NG2 cells and did not segregate with electrophysiological subtypes, indicating that AMPA/kainate receptor function represents a general feature of this cell population rather than a distinguishing characteristic.

In contrast to KA, NMDA receptor responses were observed only in a minority of NG2-glia. The proportion of responding cells was comparable to previous Ca^2+^ imaging studies in optic nerve explants (Hamilton et al., 2009), although lower than reported for corpus callosum NG2 cells (Ziskin et al., 2007). While NMDA responsiveness was not linked to general baseline membrane properties, it was associated with specific electrophysiological features as responding cells consistently lacked A-type K^+^ currents. This suggests that NMDA receptor expression is restricted to a subset of NG2 cells within the broader population. Importantly, however, NMDA responsiveness did not define a separate cell class, but rather occurred within the established NG2-glial population. One possible explanation is that NMDA receptor expression reflects differences in maturation state. As NG2 cells mature, their voltage-gated conductance change, often accompanied by increased expression of inwardly rectifying K^+^ channels (Larson et al., 2016). The membrane properties of NMDA-responsive cells observed here resemble those of more mature NG2 cells (De Biase et al., 2010), raising the possibility that NMDA receptor expression is developmentally regulated in the optic nerve. Indeed, NMDA receptor mediated currents have been detected at different rates throughout development in the forebrain as well, but according to the opposite pattern: NMDA-receptor density has been shown to peak between roughly the first 5 postnatal weeks, after which it steadily decreased (Spitzer et al., 2019). It is also possible that NMDA-evoked currents become more easily detectable in more mature NG2 cells due to changes in receptor subunit composition, receptor trafficking or receptor localization during NG2-glia maturation. The exact nature of developmental mechanisms underlying these changes remain unclear, and overall, the functional significance of NMDA receptors in NG2 cells remains unclear as well. Genetic ablation studies have shown that NMDA receptor loss does not strongly affect NG2-glial development or differentiation (De Biase et al., 2011), although modulatory roles in receptor expression have been proposed (De Biase et al., 2011). Recently, it is also suggested that NMDA receptors are important for activity-dependent myelination (Lundgaard et al., 2013; Spitzer et al., 2019). Our findings support the view that NMDA receptor function is limited to a subset of NG2 cells and does not represent a universal or defining feature of this cell type, but their expression and role is rather context- or state-dependent (Kamen et al., 2022).

Passive astrocytes in the optic nerve also exhibited functional responses to KA and NMDA, although responses to either agonist were not observed in all cells. Approximately half of passive astrocytes responded to KA, consistent with previous studies in optic nerve, thalamus, and cerebellum (Burnashev et al., 1992; Hamilton et al., 2008; Höft et al., 2014). KA responses in astrocytes were heterogeneous. In most cells, responses were consistent with activation of non-selective cationic conductance, as described in other brain regions. However, in a subset of astrocytes, KA application led to a reduction of outward K^+^ currents, a feature previously described in NG2 cells and *in vitro* astrocytes, but not in astrocytes *in situ* (Borges and Kettenmann, 1995; Muller et al., 1992; Robert and Magistretti, 1997; Steinhauser et al., 1994). This mechanism may serve to limit extracellular K^+^ accumulation during periods of high neuronal activity. The variability in KA responses likely reflects differences in receptor subunit composition, consistent with known regional and intra-regional heterogeneity of glutamate receptors in astrocytes (David et al., 1996; Droste et al., 2017; Höft et al., 2014; Iino et al., 2001). However, in the present study, response variability could not be linked to baseline electrophysiological properties, further supporting the notion that glutamate responsiveness represents an additional functional dimension rather than a defining subtype characteristic. NMDA receptor responses in astrocytes were more uniform in their kinetics and consistent with previous reports from other CNS regions (Lalo et al., 2006; Palygin et al., 2011; Schipke et al., 2001; Steinhauser et al., 1994). These responses may contribute to neuroprotective mechanisms, for example by modulating local activity through gliotransmitter release (Letellier et al., 2016). Given their close association with nodes of Ranvier, optic nerve astrocytes are anatomically well positioned to mediate such effects. Nevertheless, not all astrocytes responded to NMDA, again indicating functional heterogeneity within this population.

In summary, our findings demonstrate that AMPA/KA- and NMDA-mediated glutamate receptor responses in optic nerve glia are heterogeneous and occur in subsets of NG2 cells and astrocytes. Importantly, these responses do not define distinct cell types, but are distributed within established glial populations. This suggests that glutamatergic signaling represents a modulatory and state-dependent feature rather than a primary determinant of glial identity. Such functional diversity may enable glial cells to flexibly adapt to local neuronal activity and metabolic demands, contributing to axon–glia communication, homeostasis, and myelination in white matter. These results provide a framework for future studies addressing the dynamic roles of glial neurotransmitter signaling in health and disease.

## Data Availability

The raw data supporting the conclusions of this article will be made available by the authors, without undue reservation.
