# Supplementary material for: Diversity of glutamate receptors in glial cells of the mouse optic nerve
Source: Front Cell Neurosci. 2026 Sep 8;20:1911068. doi: 10.3389/fncel.2026.1911068 (PMC13593437; doi:10.3389/fncel.2026.1911068)
Supplement: Supplementary file 1 [file Data_Sheet_1.PDF]

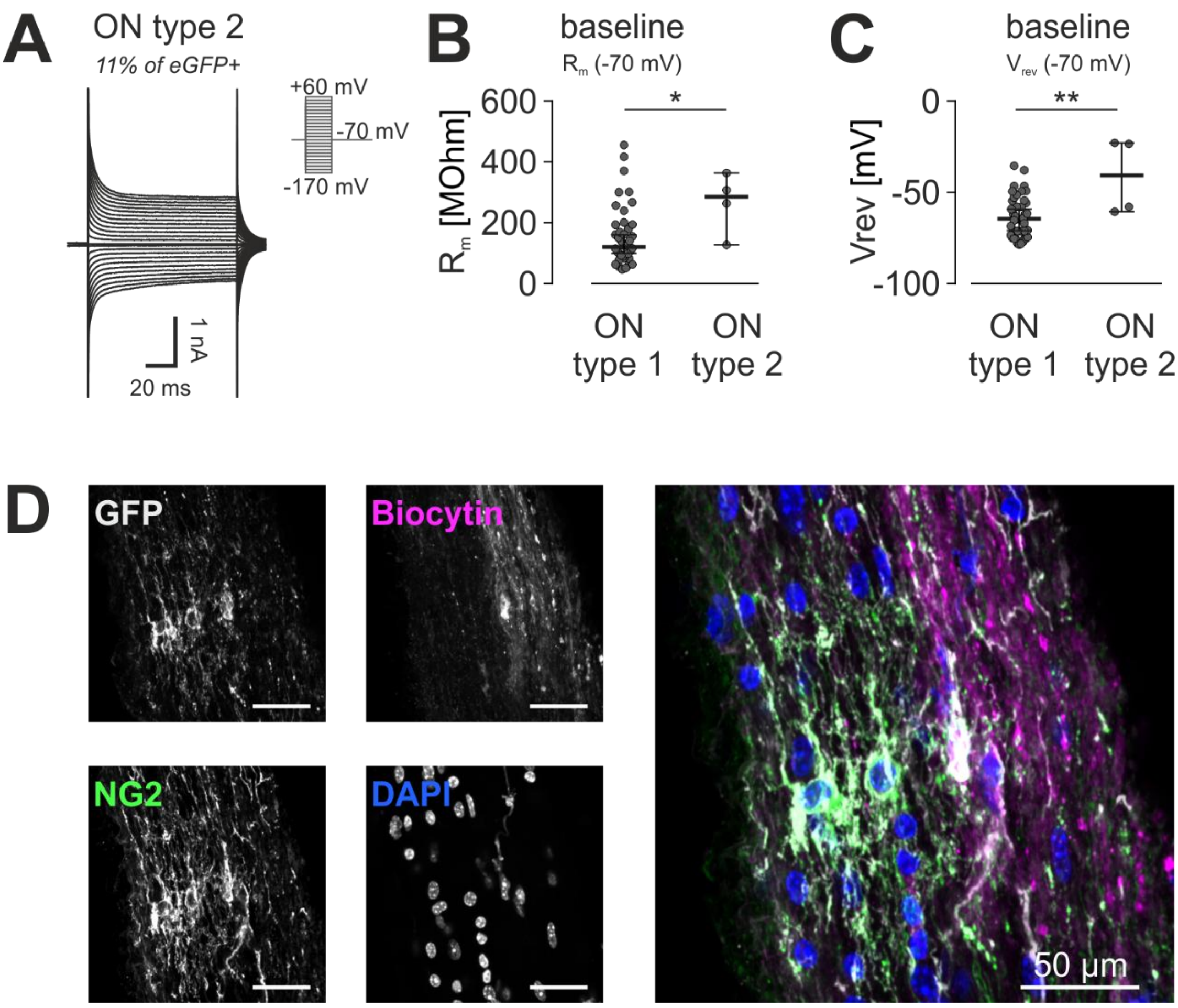

Suppl. Fig. 1. Electrophysiological properties of ON type 2 cells. (A) Representative membrane currents of ON type 2 cells (~11% of recorded cells). **(B–C)** Quantification of baseline membrane resistance and reversal potential in ON type 1 (n = 42) and type 2 cells (n = 4) which were significantly different (Mann-Whitney U tests, Bonferroni adjusted *p*-level: 0.025). **(D)** Representative image of ON stained against GFP (hGFAP-eGFP), biocytin (patched ON type 1 cell), NG2 and DAPI.

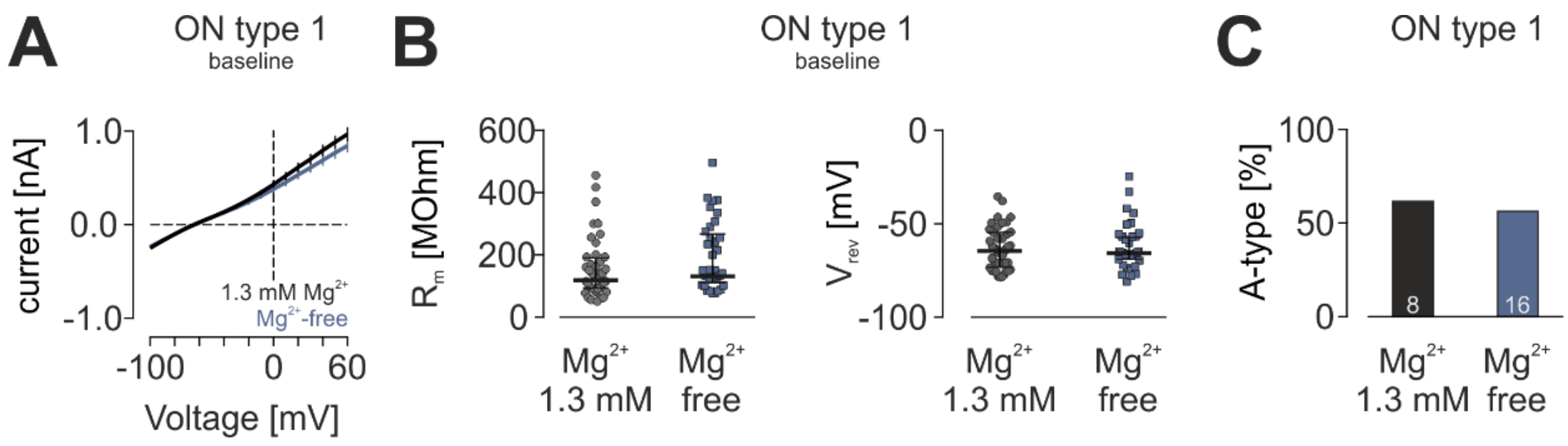

Suppl. Fig. 2. Baseline membrane properties of ON type 1 cells under Mg^{2+}-free conditions. Average I-V relationships (A), quantification of membrane resistance (left) and reversal potential (right, **B**) and expression of A-type K^{+} currents (**C**) in **ON type 1** cells under Mg^{2+}-containing (1.3 mM) and Mg^{2+}-free conditions.

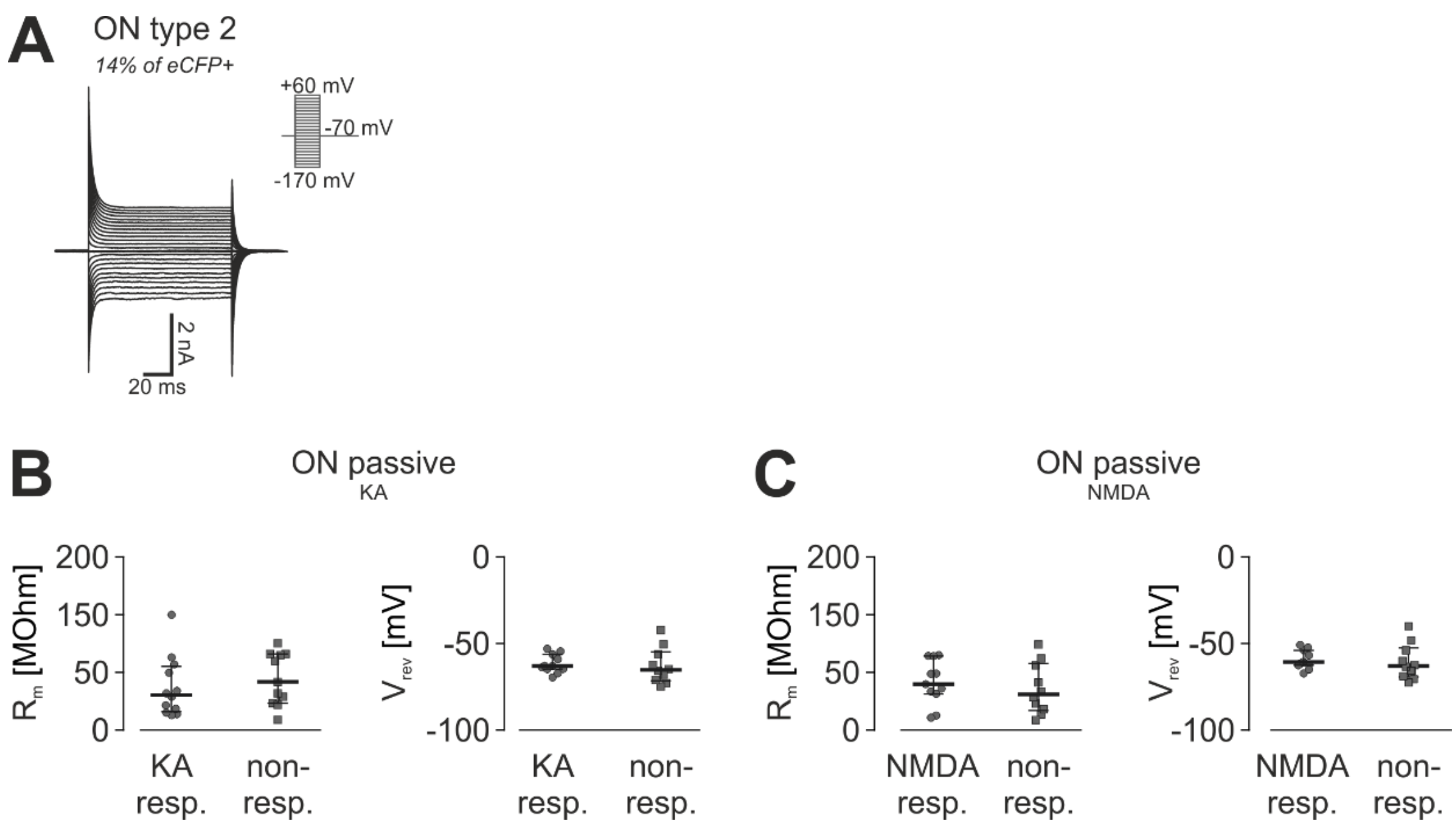

Suppl. Fig. 3. Additional information on ON Cx43-ki-eCFP+ cells. **(A)** Representative membrane currents of eCFP+ ON type 2 cells in response to voltage steps (−170 mV to +60 mV), illustrating their characteristic time- and voltage-dependent current profile. ON type 2 cells represent the minority (~14%) of eCFP+ cells. **(B)** Quantification of membrane resistance (left) and reversal potential (right) showing no differences in KA-responding and non-responding **ON type 1** cells. **(C)** Same analysis as in (B),but for NMDA- responding and non-responding ON passive cells.
